# Spironolactone in cardiovascular disease: an expanding universe?

**DOI:** 10.12688/f1000research.11887.1

**Published:** 2017-09-22

**Authors:** John W. Funder

**Affiliations:** 1 Hudson Institute, Monash Medical Centre and Monash University, 27-31 Wright St Clayton, VIC, 3168, Australia

**Keywords:** spironolactone, primary aldosteronism, heart failure, resistant hypertension

## Abstract

Spironolactone has been marketed for over half a century as a ‘potassium-sparing diuretic’, used primarily in patients with ascites. With the realization that primary aldosteronism is the most common (5-13%) form of secondary hypertension, it has become widely used as a mineralocorticoid receptor antagonist. More recently, in the wake of the RALES trial, spironolactone in addition to standard therapy has been shown to be very beneficial in heart failure with a reduced ejection fraction. Despite the failure of the TOPCAT trial, spironolactone is being increasingly used in diastolic heart failure (i.e. with a preserved ejection fraction). The third currently accepted role for spironolactone is in hypertension resistant to three conventional antihypertensives including a diuretic, where it has been proven to be effective, in contra-distinction to renal artery denervation. Finally, brief consideration will be given to ‘areas in waiting’ – pulmonary hypertension/fibrosis, cancer – where spironolactone may play very useful roles.

## Introduction

Spironolactone (Aldactone ^R^) is the prototypic mineralocorticoid receptor antagonist (MRA), patented over 50 years ago and still in widespread clinical use. Structurally, it is a synthetic derivative of progesterone, based on the knowledge that physiologically (e.g. in pregnancy) progesterone is a potent MRA. It shares progesterone receptor agonist activity with its parent steroid, and in addition is an androgen receptor antagonist. A second generation MRA (eplerenone) is less potent, much more selective, more expensive to make, and much more expensive to buy, except in Japan. Currently third and fourth generation MRAs are in development – as potent as spironolactone, as selective as eplerenone, non-steroidal, cheap to manufacture and (hopefully) with a long patent life; fourth generation are as above, but renal tubule sparing (lessening the chance of hyperkalemia) or otherwise tissue-selective.

This brief review will focus on established indications for spironolactone use – primary aldosteronism (PA), heart failure, resistant hypertension (ReHT) – in cardiovascular disease. As noted in a timely publication on emerging cardiovascular indications for MRAs ^1^, there are preclinical and clinical data supporting the potential efficacy of MRAs in pulmonary arterial hypertension, pulmonary fibrosis, arrhythmias, valvular heart disease, renal disease and stroke – though all fall short of being licensed, in this litigious age, for use. Accordingly, the main focus of the review will be on the three main areas listed above, with a more cursory discussion of some of the potentially expanding universe.

## Primary Aldosteronism

If you were taught that PA was a rare (~1%) and relatively benign form of hypertension, you, (and I) were misled: we now know PA to be ~ an order of magnitude more common, and with a higher cardiovascular risk profile, than essential hypertension ^2, 3^. Current estimates put prevalence at 5–13% of unselected hypertensives, the variance reflecting adoption of strict or relaxed ‘cut-offs’ for plasma aldosterone concentration (PAC) and plasma renin levels, measured as activity or concentration. Both historically ^4^ and over the past decade there are emerging data that the prevalence of ‘inappropriate aldosterone secretion’ or ‘dysregulated aldosterone secretion’ may take the prevalence from ~10% to ~50% in hypertensives ^5– 8^, and ~15% in normotensives ^9^. A minority of hypertensives (3–5%) have PA due to a unilateral adrenal adenoma, or much less commonly unilateral hyperplasia, which is lateralized by adrenal venous sampling: the expanding majority appear to have bilateral adrenal hyperplasia. Treatment for unilateral disease is laparoscopic adrenalectomy: that for bilateral disease, or partial success of surgery, is not surprisingly an MRA, plus additional anti-hypertensives if/as required to normalize blood pressure levels.

Spironolactone is the MRA in common use in PA, and should be used at low dose (12.5–50 mg/day) to minimize side effects and optimize compliance. The low dose can be combined with amiloride, which targets the epithelial sodium channel to shed sodium, not the mineralocorticoid receptor (MR). Inappropriate sodium status is key to the deleterious effects of aldosterone in PA: in chronic salt deficiency even higher levels of aldosterone are homeostatic, with no deleterious effects on the cardiovascular system. Persistence of hypertension after biochemical cure of an APA (normal plasma [K+], PAC, renin) is commonly attributed to underlying essential hypertension, or established vascular damage; in either circumstance a case can be made for the careful (i.e. after normalization of PAC, sometimes depressed post-operatively) addition of a low dose of MRA. In early clinical studies ^10^ Jeunemaitre and colleagues showed that spironolactone (mean dose 98 mg/day) given to patients with essential hypertension lowered blood pressure by 18/9 mmHg, with an increase in average plasma [K+] of 0.7 meq/L. In a subsequent treatment to effect study on essential hypertensives where patients were given eplerenone (50/100/200 mg/day – i.e. doses equivalent to or less than that of spironolactone), blood pressure fell to a similar extent, but plasma [K+] was on average elevated by only ≤0.2 meq/L ^11^. Experimental studies on DOC/salt rats divided into 5 groups (control; DOC/salt for 4 weeks; DOC/salt for 8 weeks; DOC/salt for 4 weeks, DOC withdrawn weeks 5–8; DOC/salt for 8 weeks, eplerenone given weeks 5–8) directly demonstrated the role of MRAs in reversing tissue damage ^12^. DOC/salt raised blood pressure and cardiac fibrosis progressively, with levels at 8 weeks ~ twice those at 4 weeks. Withdrawal of DOC over weeks 5–8 showed levels of cardiac collagen and NAD(P)H expression in coronary vessels equivalent to those in rats killed after 4 weeks of DOC/salt. In contrast, animals continuing on DOC/salt receiving eplerenone from weeks 5–8 showed cardiac fibrosis and coronary inflammatory markers reduced to control levels, evidence that MRAs can not only lower blood pressure but also reverse established tissue damage.

Spironolactone has active metabolites with a long (~18–24 hours) half-life; eplerenone has no active metabolites, and a much shorter half-life. It has a much lower (2–3%) affinity for MR than spironolactone, but is much less plasma bound. Overall, eplerenone has ~ half the potency of spironolactone, but appears less prone to cause hyperkalemia (cf 10, 11), presumably reflecting its much more rapid clearance. Generation 4 MRAs, aimed at being renal tubule-sparing to minimize hyperkalemia in heart failure, are probably in fact dangerous in PA: hypokalemia is more life-threatening than hyperkalemia. So-called low renin hypertension (a description, not a diagnosis) includes a majority of patients without PA on present criteria but who show identical responses – lowering of blood pressure and left ventricular mass index as PA patients when both are treated with low-dose spironolactone ^13^, consistent with their constituting a forme fruste of PA. The same is true for ReHT, to be considered in detail later in the review: such patients respond equally well to spironolactone whether or not they have PA (15–20%) on current criteria. Perhaps in muted recognition, the 2016 guideline from the Endocrine Society ^14^ recommends that patients at risk of PA on clinical grounds might have an MRA included in their drug treatment even if they are negative for PA on screening or subsequent confirmatory/exclusion testing.

## Heart failure

Until two decades ago, MR were not on the cardiologic horizon, and aldosterone just one of the ‘neurohumoral’ factors commonly acknowledged en bloc and often essentially dismissed as of secondary importance. This changed in 1999, with the publication of RALES (the Randomized Aldactone Evaluation Study: ^15^) in progressive New York Heart Association stage III congestive heart failure. Patients were randomized into standard-of-care (ACE inhibitors, beta-blockers, diuretics etc.) alone, or plus low-dose spironolactone. The daily starting dose was one tablet (25 mg), to be titrated up or down on clinical grounds: the mean daily dose over the course of the study was 26 mg/day. Recruitment stopped half-way through, reflecting the major differences in outcome between the two arms – a 30% reduction in mortality, and a 35% lower level of hospitalization; the data were so stark that not only cardiologists but even hospital administrators took notice. The findings were also hailed as evidence that spironolactone was blocking the effect of aldosterone on the heart via ‘the aldosterone receptor’.

This has subsequently been shown to be not the case, on all three counts. If you believe in evolution, the MR is clearly present in both cartilaginous and bony fish ^16^, millions of years before the emergence of aldosterone in lungfish, the species marking transition from the obligate aqueous to the terrestrial environment. The presumptive ligand for these MR is cortisol, which has equivalent high affinity as aldosterone for human MR. In humans ~90% of MR are normally cortisol-occupied, so that in a sense even MR may be challenged as a name: some justification might be that when the renal specificity-conferring mechanisms in the kidney tubule are blocked ^17^ or deficient ^18^ cortisol mimics aldosterone to produce a classical mineralocorticoid response. MR were initially called type I corticosteroid receptors (to distinguish them from type II, now called glucocorticoid receptors): MR is now widely accepted; ‘aldosterone receptor’, however, is clearly untenable.

Secondly, what happened in RALES was not that spironolactone was blocking aldosterone: despite some patients being on diuretics, the mean PAC were in the low normal range. As noted previously, cortisol has the same high affinity for MR as aldosterone, but circulates at ~1000 fold higher levels, offset to some extent to ~100 fold higher by more extensive plasma binding. Cardiomyocytes do not express 11BHSD2, the enzyme conferring aldosterone specificity in activating epithelial MR: cardiac MR are thus overwhelmingly occupied by cortisol. Under normal circumstances cortisol in cardiomyocyte MR clearly does not mimic aldosterone; in conditions of tissue damage, reactive species generation and redox change, however, cortisol becomes an MR agonist, mimicking aldosterone ^19^. The effect of spironolactone in RALES is on cortisol-occupied (and activated, in class III heart failure) MR in stressed cardiomyocytes.

Third, if cardiomyocyte MR are overwhelmingly occupied by cortisol, an MR agonist under conditions of tissue damage, how is it that the very modest mean dose of spironolactone (26 mg/day) could have the effect it did? There would appear to be at least two factors in play. First, reflecting the long half-time of the active metabolites of spironolactone, steady-state levels take 8–10 days to achieve, at levels considerably higher than the maximum obtained with a single dose. Secondly, and probably equally if not more importantly, spironolactone is an inverse agonist, and not just a ‘blocker’ of agonist binding to MR. We commonly think of antagonists as passive blockers, binding to and sitting in the receptor and thus denying hormone access: this has been crystallized in the nomenclature of angiotensin receptor blockers. What an inverse agonist does is to add an extra dimension, by having a life of its own, and by its ability to induce intra-cellular messages opposing those of the hormone it is antagonizing. To do this the inverse agonist does not have to occupy all of the receptors – just enough for its messages to counteract those of the regular agonist hormone.

In the ischemia-reperfusion Lagendorf isolated heart preparation ^19^, cortisol and aldosterone (at equal doses) increase area-at-risk and infarct size; cortisol (and aldosterone) is antagonized by spironolactone, but not by the glucocorticoid receptor/progesterone receptor antagonist RU38486 (mifepristone). In Lagendorf preparations from adrenalectomized rats, in the absence of endogenous corticosteroids, spironolactone reduces area-at-risk and infarct size, thus the designation as an inverse agonist. In epithelia spironolactone may block aldosterone – given its very low concentration – accessing and activating MR: in the cardiomyocyte, much higher levels of cortisol make mere ‘blockade’ a tall order, bringing into play the ability of spironolactone to occupy a proportion of cardiomyocyte MR and produce its beneficial effects as an inverse agonist.

RALES patients had relatively low (‘reduced’) ejection fractions, so-called HFrEF: low-dose spironolactone is now commonly accepted to be worthwhile, as long as the treating physician withdraws potassium supplements, and monitors plasma [K+], particularly in the elderly or those with reduced renal function. The data on diastolic heart failure, with a preserved ejection fraction (HFpEF), were complicated by the so-called TOPCAT trial sponsored by the National Institutes of Health ^20^. The problem with the study, which overall reported no utility for spironolactone in HFpEF, was a dichotomy between the patients entered in the Western Hemisphere (USA/Brazil/Canada/Argentina) and those from Russia and Georgia. In brief, it appears that most of the patients enrolled from the latter centres were not in heart failure, as evidenced by the very low event rate in the control (no spironolactone) group, with in consequence no significant effect of the added drug in the spironolactone treated group.

By several indices other than the primary outcome measures the patients from the West showed the utility of addition of low-dose spironolactone. Subsequent studies ^21, 22^, admittedly in smaller groups of patients, in China and Mexico have clearly shown the benefit of low-dose spironolactone in HFpEF. In addition, in a recent transnational review, the authors – including the highly experienced trialist Bertram Pitt, lead investigator on RALES and TOPCAT – concluded that “spironolactone should be considered for HFpEF patients with elevated natriuretic peptides...and structural abnormalities on echocardiography”. In a manuscript gently entitled “Tailoring mineralocorticoid receptor antagonist therapy in heart failure patients: are we moving to a personalized approach?” ^23^, they cite “challenging inconsistencies” in trials of HFpEF: indeed. TOPCAT might be remembered as half-dead-cat: no bounce.

## Resistant hypertension

In addition to PA and heart failure, the best accepted role for spironolactone in clinical cardiovascular practice is in ReHT. ReHT is defined as hypertension persisting despite the established use of 3 conventional (sic) antihypertensives including a diuretic. For a diagnosis of ReHT white-coat hypertension needs to be excluded, ideally by 24 hour monitoring, as does irregularity in or failure of compliance: the incidence of ReHT, excluding the above confounders, is of the order of 12–15% of hypertension. For some time renal denervation was proposed as therapy: a recent meta-analysis of trials of renal denervation, with or without sham operated controls, found no resolution of elevation blood pressure, thus comprehensively rejecting this proposition ^24^.

In contrast, a recent systematic meta-analysis showed that MRAs reduce blood pressure more effectively than other fourth line agents (Bisoprolol, Doxazosin, Furosemide, additional RAS blockade), with falls of 7.4–11.9 mmHg more than that seen with active comparator ^25^. In a subsequent meta-analysis from China ^26^ ReHT patients receiving spironolactone showed a 16.7/6.1 mmHg lowering of systolic/diastolic blood pressure compared with placebo. In a second meta-analysis from China ^27^, a splendidly novel collaboration between cardiology and civil engineering, findings were similar to both the above trials. Against placebo, spironolactone treated ReHT patients showed a 15.7/6.2 mmHg fall in systolic/diastolic office blood pressure, and 8.7/4.1 mmHg in ambulatory home measurements. In comparison with alternative drugs (beta-blocker, candesartan, alpha methyldopa) spironolactone reduced home systolic blood pressure by 4.5 mmHg. The conclusion reached by all three meta-analyses is that the addition of spironolactone provides a beneficial effect on blood pressure in patients with ReHT.

The case for the addition of spironolactone – or, for the rich, eplerenone – to their previous medication regime appears very strong for patients with ReHT. Three final comments are as follows. First, one group of patients included in the Endocrine Society Guideline ^8^ for the Management of Primary Aldosteronism are those with blood pressure ‘controlled’ (<140/90) on four or more antihypertensives drugs: such patients may well benefit by replacing the beta-blocker, for example, with low-dose spironolactone. Second is what is measured in all the studies subjected to meta-analysis i.e. blood pressure: what is not measured directly is the effect of spironolactone on the vasculature, which may be an equal factor in reducing morbidity and mortality – such studies have not been done, and given that spironolactone is decades out of patent, probably never will be. Finally, as previously cited, the ~85% of ReHT patients who do not appear to have PA on the current criteria and the ~15% who do respond indistinguishably to the addition of spironolactone: Ockham’s razor would say that in the first instance perhaps the current criteria for PA are rather too much on the strict side.

The above sections cover the currently accepted indications for spironolactone in cardiovascular disease. Among the almost myriad possibilities for possible therapeutic roles cited in the introduction ^1^ there are two – pulmonary hypertension/fibrosis that will be briefly surveyed, and at the end of the essay a sleeper – or in racing terms, an outsider.

The pulmonary effects of spironolactone were explored over fifty years ago ^28^; more recently, work towards an evidence base for efficacy has focused predominantly on experimental animals and *in vitro* studies. In two models of pulmonary hypertension (PH) in mice (hypoxia, prevention model; monocrotaline-induced, prevention and treatment model) spironolactone attenuated a series of effects in terms of prevention – increase in right ventricular systolic pressure, pulmonary arterial muscularization, right ventricular fibrosis, pulmonary vascular resistance. In treatment of established PH, spironolactone decreased right ventricular systolic pressure and pulmonary vascular resistance ^29^. Studies on rodent models subjected to bleomycin-evoked pulmonary fibrosis showed that both spironolactone and eplerenone were efficacious in attenuating pulmonary fibrosis ^30^; a parallel study from China confirmed that spironolactone attenuated bleomycin-induced acute pulmonary injury and fibrosis, in part via inhibition of MR-mediated circulating monocyte and alveolar macrophage phenotype switching ^31^. Given the clinical difficulties in managing PH patients, it would seem that the time might be ripe for a major, well-constructed clinical trial in patients with established disease.

The sleeper is the role of spironolactone, in and outside cardiovascular disease, in cancer. Anthracycline group chemotherapeutic agents are used post-surgery in breast cancer patients, and are known to induce cardiomyopathy. In an elegant study from Turkey ^32^, 83 patients were divided into a group receiving spironolactone (n=43), and a control group (n=40). In the control group left ventricular ejection fraction declined from 67.7±6.3 to 53.6±68 (p<0.001); in the group receiving spironolactone the equivalent figures were 67.0±6.1 to 65.7±7.4 (p=0.094). The difference between spironolactone and control was, not surprisingly, highly significant (p<0.001).

Extending this study, another group from Turkey used rats to study the cardiovascular toxicity induced by concomitant trastuzumab and thoracic radiotherapy, given their awareness of the first clinical study. They showed that acutely the combination did not affect cardiac inflammation and fibrosis scores, or TGFβ expression; chronically, however, spironolactone significantly attenuated fibrosis (p<0.004) and TGFβ expression (p<0.002) compared with trastuzumab and radiotherapy alone ^33^.

Finally, the sleeper in terms of spironolactone and cancer is a very recent study entitled ‘Spironolactone Use and the Risk of Incident Cancers: a Retrospective, Matched Cohort Study” ^34^. Patients exposed to spironolactone between 1986 and 2013 (n=74,272) were matched 1:2 with unexposed controls. Prespecified primary outcomes were the first incidence of ovarian, endometrial, pancreatic, colorectal, prostate, renal cell, pharyngeal and thyroid cancers, and myleomonoblastic/-cytic leukemias; secondary outcomes were the remaining 27 types of cancer. The results were that there is no evidence for an increased risk of any cancer associated with the use of spironolactone. There was, however, strong evidence for a significantly lower risk of prostate cancer (hazard ratio 0.69: 95% confidence limits 0.60–0.80; p<0.001). As the authors conclude, “The possible mechanisms and clinical implications merit further investigation.” Quite so.

## Envoi

Spironolactone has been the workhorse MRA for over fifty years. It is probable that additional therapeutic roles for MRAs will be established, and third and fourth generation agents will be developed to address particular organs or issues selectively. Inevitably these will be expensive, to cover costs of development and marketing, and will incur push-back from insurers and governments in terms of reimbursability. These advances in targets and medication notwithstanding, it is probable that spironolactone, carefully used and at relatively low doses, will be around for another fifty years.
